# Aberrant DNA methylation profiles of inherited and sporadic colorectal cancer

**DOI:** 10.1186/s13148-015-0165-2

**Published:** 2015-12-21

**Authors:** Nora Sahnane, Francesca Magnoli, Barbara Bernasconi, Maria Grazia Tibiletti, Chiara Romualdi, Monica Pedroni, Maurizio Ponz de Leon, Giulia Magnani, Luca Reggiani-Bonetti, Lucio Bertario, Stefano Signoroni, Carlo Capella, Fausto Sessa, Daniela Furlan, AIFEG

**Affiliations:** Department of Surgical and Morphological Sciences, Section of Anatomic Pathology, University of Insubria, Via O. Rossi, 9, 21100 Varese, Italy; Department of Pathology, Ospedale di Circolo, Varese, Italy; CRIBI Biotechnology Center, University of Padova, Padua, Italy; Department of Diagnostic Medicine, Clinical and Public Health, University of Modena and Reggio Emilia, Modena, Italy; Department of Pathology, Policlinico di Modena, Modena, Italy; Unit of Hereditary Digestive Tract Tumours, Fondazione IRCCS—Istituto Nazionale dei Tumori Milan, Modena, Italy

**Keywords:** Colorectal cancer, LINE-1, Gene hypermethylation, Lynch syndrome, Microsatellite instability, Early onset colorectal cancer

## Abstract

**Background:**

Aberrant DNA methylation has been widely investigated in sporadic colorectal carcinomas (CRCs), and extensive work has been performed to characterize different methylation profiles of CRC. Less information is available about the role of epigenetics in hereditary CRC and about the possible clinical use of epigenetic biomarkers in CRC, regardless of the etiopathogenesis. Long interspersed nucleotide element 1 (LINE-1) hypomethylation and gene-specific hypermethylation of 38 promoters were analyzed in multicenter series of 220 CRCs including 71 Lynch (Lynch colorectal cancer with microsatellite instability (LS-MSI)), 23 CRCs of patients under 40 years in which the main inherited CRC syndromes had been excluded (early-onset colorectal cancer with microsatellite stability (EO-MSS)), and 126 sporadic CRCs, comprising 28 cases with microsatellite instability (S-MSI) and 98 that were microsatellite stable (S-MSS). All tumor methylation patterns were integrated with clinico-pathological and genetic characteristics, namely chromosomal instability (CIN), *TP53* loss, *BRAF*, and *KRAS* mutations.

**Results:**

LS-MSI mainly showed absence of extensive DNA hypo- and hypermethylation. LINE-1 hypomethylation was observed in a subset of LS-MSI that were associated with the worse prognosis. Genetically, they commonly displayed G:A transition in the *KRAS* gene and absence of a CIN phenotype and of *TP53* loss. S-MSI exhibited a specific epigenetic profile showing low rates of LINE-1 hypomethylation and extensive gene hypermethylation. S-MSI were mainly characterized by *MLH1* methylation, *BRA*F mutation, and absence of a CIN phenotype and of TP53 loss. By contrast, S-MSS showed a high frequency of LINE-1 hypomethylation and of CIN, and they were associated with a worse prognosis. EO-MSS were a genetically and epigenetically heterogeneous group of CRCs. Like LS-MSI, some EO-MSS displayed low rates of DNA hypo- or hypermethylation and frequent G:A transitions in the *KRAS* gene, suggesting that a genetic syndrome might still be unrevealed in these patients. By contrast, some EO-MSS showed similar features to those observed in S-MSS, such as LINE-1 hypomethylation, CIN, and *TP53* deletion. In all four classes, hypermethylation of *ESR1*, *GATA5*, and *WT1* was very common.

**Conclusions:**

Aberrant DNA methylation analysis allows the identification of different subsets of CRCs. This study confirms the potential utility of methylation tests for early detection of CRC and suggests that LINE-1 hypomethylation may be a useful prognostic marker in both sporadic and inherited CRCs.

**Electronic supplementary material:**

The online version of this article (doi:10.1186/s13148-015-0165-2) contains supplementary material, which is available to authorized users.

## Background

Aberrant DNA methylation, including both widespread demethylation as well as site-specific gene hypermethylation, deregulates the genome and contributes to the loss of tissue homeostasis observed in aging and in cancer. Changes in DNA methylation have been widely investigated in sporadic colorectal carcinoma (sCRC), and extensive work has been carried out both to characterize different methylation profiles of colorectal carcinoma (CRC) [1–4] and to investigate the possible clinical applications of epigenetic biomarkers for the early detection of CRC, risk assessment, prognostication, and therapeutic opportunities [5–8].

Recently, whole-genome methylation analyses of CRCs, precursor lesions, and normal colorectal mucosa provided confirmation that aberrant DNA methylation is common in CRCs and occurs early in colorectal tumorigenesis. Cancer-specific de novo methylation has been detected in aberrant crypt foci [9, 10] as well as being extensively observed in the histologically normal colonic mucosa of patients predisposed to multiple serrated polyps, the proposed precursors of CRC with a CpG island methylator phenotype-high (CIMP-H) [11]. To date, it is widely accepted that CIMP-H is a distinct form of epigenomic instability in sporadic CRC [2, 12] which is strongly associated with a hypermutated profile [13], with *BRAF*^*V600E*^ mutation [14] and with microsatellite instability (MSI) through epigenetic silencing of MLH1 [15, 16].

Genome-wide-DNA hypomethylation is the other early epigenetic alteration that has been observed in sporadic CRCs [17–19], and it has been associated with genomic and chromosomal instability (CIN) [20–23], as well as with the deregulation of gene transcription and activation of retrotransposons [24]. Recently, long interspersed nucleotide element 1 (LINE-1) hypomethylation has been recognized as an independent factor for increased cancer-related mortality and overall mortality in CRC patients [25–27]. In addition, some recent studies proposed this biomarker for familial cancer risk assessment, suggesting that LINE-1 hypomethylation is one of the distinguishing features of non-Lynch Syndrome familial CRC [28, 29] and that it is associated with early-onset CRC [28, 30].

However, until now, the role of epigenetics in hereditary and familial CRC has not been thoroughly explored, and its contribution toward carcinogenesis has not been characterized because accurate cancer genetics risk assessments are often lacking in the familial cases analyzed [30–32]. On the other hand, the evaluation of aberrant DNA methylation patterns in well-characterized inherited CRCs compared with those observed in sporadic CRCs could improve our knowledge of general mechanisms of epigenetics in colorectal carcinogenesis and help to identify common biomarkers for cancer risk assessment and for prognostication.

For this purpose, we determined both widespread hypomethylation as well as site-specific gene hypermethylation in a large and multicenter tumor series including 71 Lynch (Lynch syndrome (LS)) CRCs with an identified pathogenic germline mutation, 23 early onset CRCs (under 40 years) in which the main inherited CRC syndromes had been excluded, and 126 sporadic CRCs. All tumor methylation patterns were integrated with clinico-pathologic profiles and genetic characteristics, namely MSI and CIN status, *TP53* loss and *BRAF*, and *KRAS* mutations.

## Results

### Patient grouping, genetic, and clinico-pathologic evaluation

Formalin-fixed and paraffin-embedded (FFPE) CRCs were collected from three Italian institutes and included (I) 71 CRCs showing MSI from Lynch patients (Lynch colorectal cancer with microsatellite instability (LS-MSI); ORPHA 144) carriers of mismatch repair (MMR) germline mutations including 44 MLH1, 22 MSH2, 4 MSH6, and 1 EPCAM pathogenetic variants. In this subset of cases, only class 5 variants were considered as defined by International Society for Gastrointestinal Hereditary Tumors (InSiGHT) Variant Interpretation Committee (Mismatch Repair Gene Variant Classification Criteria, Version 1.9 August 2013); (II) 28 sporadic CRCs showing high microsatellite instability (S-MSI); (III) 98 sporadic CRCs without MSI (S-MSS). For sporadic cases, previously characterized for MSI, the presence of known hereditary cancer syndromes was excluded; (IV) 23 microsatellite stability (MSS) CRCs from patients younger than 40 years (early-onset colorectal cancer with microsatellite stability: EO-MSS) recruited through the specialized Colorectal Cancer Registry of Modena in the period 1984–2008. As recently reported by Magnani G et al. [33], FAP (familial adenomatous polyposis; ORPHA 733), MAP (MYH associated polyposis; ORPHA 247798), and LS were excluded in these 23 cases, as well as specific clinico-pathologic, genetic, and epigenetic features were examined. The clinico-pathological data are summarized in Table 1. Mean age of patients at diagnosis was 59.6 years. By definition, all EO-MSS were 40 years old or younger, whereas patients with both S-MSI and S-MSS were significantly older (mean value 70 years). The average age of CRC onset in LS patients was in the mid-late 40s (47.1 years), decades younger than that observed in the sporadic cohort. In all analyzed subsets, male patients were more numerous than females. According to the site, nearly all (93 %) S-MSI occurred proximal to the splenic flexure, as well as the majority (69 %) of LS-MSI. On the contrary, S-MSS and EO-MSS mainly affected the distal colon (72 and 87 % of the cases, respectively). According to the histopathological variants, mucinous adenocarcinomas defined as tumors with more than 50 % of the lesion being composed of pools of extracellular mucin prevailed in S-MSI patients (18 out of 28 cases; 64 %) and were progressively less represented in LS-MSI, EO-MSS, and S-MSS (31, 13, and 7 % of the cases, respectively). A subset of unstable cases (15 % of S-MSI and 12 % of LS, respectively) and 4 S-MSS (4 %) displayed signet ring cell differentiation, defined by the presence of more than 50 % of neoplastic cells with prominent intracytoplasmatic mucin and nuclear displacement. Finally, as expected, medullary carcinoma characterized by sheets of neoplastic cells associated with prominent infiltration by intraepithelial lymphocytes, was observed only in the MSI cohort (29 % of S-MSI and 12 % of LS-MSI, respectively). Low-intermediate and high-grade adenocarcinomas were equally represented both in LS-MSI and S-MSI, with a prevalence of “tumor,” “nodes,” “metastasis” (TNM) stages I and II (72 %). Conversely, most EO-MSS (87 %) and S-MSS (85 %) were low-intermediate grade neoplasms which were frequently diagnosed at advanced stages (64 and 59 % of the cases, respectively).

**Table 1:** Main clinico-pathologic characteristics of CRCs

	LS-MSI	S-MSI	S-MSS	EO-MSS
	No. of tumors/total^a^ (%)			
Age				
- Mean, years	47.1	70.5	69.5	35.4
- Range, years	30–78	41–88	41–91	24–40
Gender				
- Female	33/71 (47)	10/28 (36)	41/94 (44)	7/23 (30)
- Male	38/71 (53)	18/28 (64)	53/94 (56)	16/23 (70)
Site				
- Proximal colon	36/52 (69)	26/28 (93)	27/98 (28)	3/23 (13)
- Distal colon	16/52 (31)	2/28 (7)	71/98 (72)	20/23 (87)
Histological type				
- Mucinous	21/67 (31)	18/28 (64)	7/98 (7)	3/23 (13)
- Medullary	8/67 (12)	8/28 (29)	0/98 (0)	0/23 (0)
- Signet ring cell	8/67 (12)	4/28 (15)	4/98 (4)	0/23 (0)
Tumor grade				
- G1, G2	30/62 (48)	13/28 (46)	83/98 (85)	20/23 (87)
- G3	32/62 (52)	15/28 (54)	15/98 (15)	3/23 (13)
TNM stage				
- I–II	48/67 (72)	18/25 (72)	38/93 (41)	8/22 (36)
- III–IV	19/67 (28)	7/25 (28)	55/93 (59)	14/22 (64)
Follow-up				
- Alive	39/53 (74)	22/26 (85)	50/94 (53)	15/23 (68)
- Died of disease	14/53 (26)	4/26 (15)	44/94 (47)	7/23 (32)

*LS-MSI* lynch syndrome CRC, *S-MSI* sporadic MSI CRC, *S-MSS* sporadic MSS CRC, *EO-MSS* early onset CRC

^a^Clinico-pathological characteristics are not available for all cases in each subset

### LINE-1 hypomethylation profiles

**Fig. 1:**
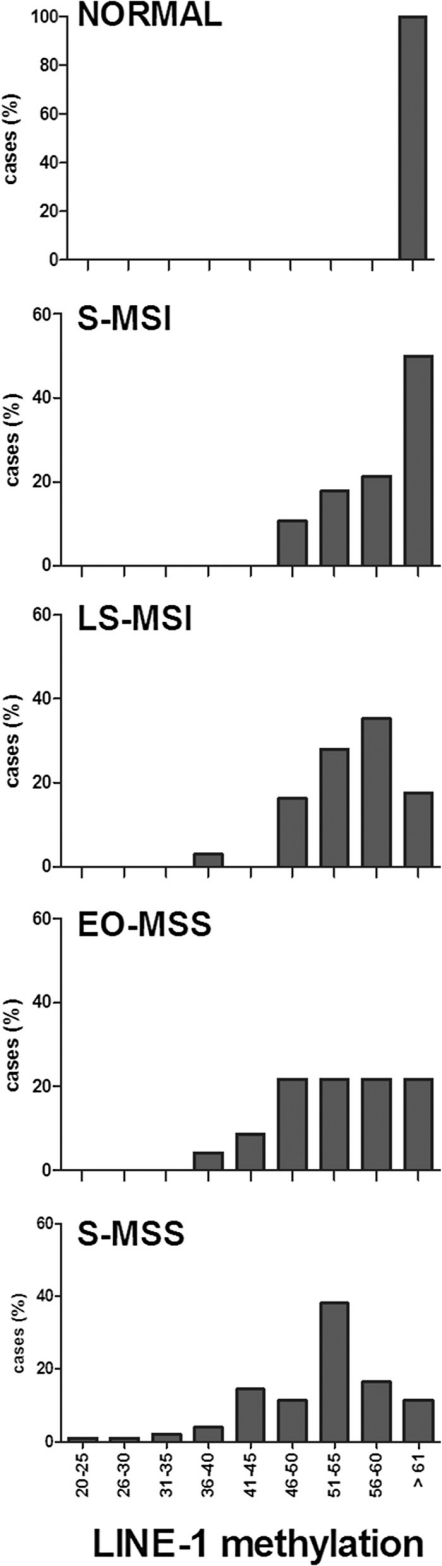
LINE-1 methylation distribution in 25 samples of normal colonic mucosa and in 217 CRCs divided in four classes: S-MSI, LS-MSI, EO-MSS, and S-MSS. The distribution of LINE-1 methylation levels and the percentages of cases are shown on *x*-axis and *y*-axis, respectively

LINE-1 methylation analysis was possible in 217 out of 220 CRCs and in all the 25 normal mucosa samples included in the study. In morphologically normal mucosa, the percentage of LINE-1 methylation was always higher than 62 % (average 64.5 ± 2 %) while in CRCs, it ranged from 24 to 68 % (average 54.3 ± 7.5 %). Distribution of LINE-1 methylation levels in the four subsets of tumors showed a significant decrease of LINE-1 methylation rate going from S-MSI to S-MSS CRC (average 59.4 ± 5.6 % versus 51.7 ± 8 %, respectively, *p* < 0.001), while intermediate methylation levels were observed in LS-MSI and in EO-MSS CRC (average 56.1 ± 5.6 % and 54.2 ± 7.6 %, respectively) (Fig. 1).

**Fig. 2:**
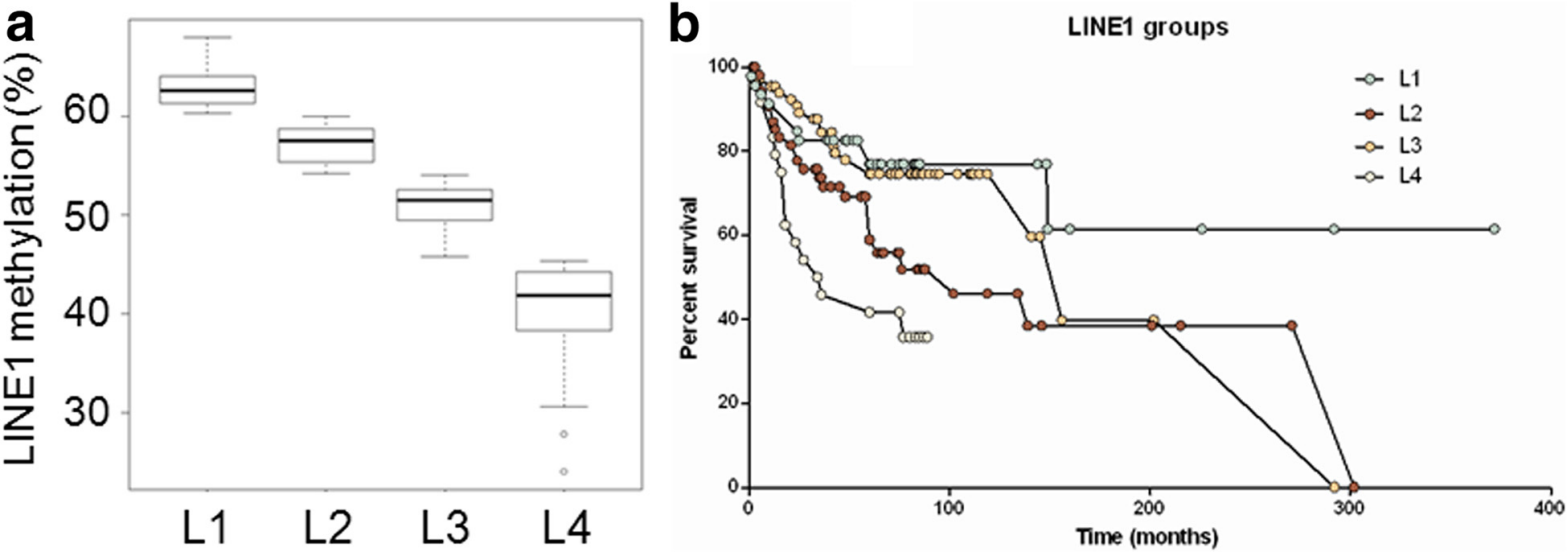
**a** Supervised clustering analysis with k-means algorithm identifies four clusters showing significant differences of LINE-1 methylation levels: L1 (51 CRCs, mean 63.1 %), L2 cluster (63 CRCs, mean 57.2 %), L3 cluster (77 CRCs, mean 50.9 %), and L4 cluster (26 CRCs, mean 40.2 %); **b** Kaplan-Meier curves showing significantly different clinical outcome in patients stratified by tumor LINE-1 methylation groups (*p* = 5 × 10^−4^)

In order to consider LINE-1 methylation as a discrete variable, we applied the k-means algorithm using a supervised clustering analysis. This method clearly subdivided all tumors into four groups showing significant differences of LINE-1 methylation levels: L1 cluster (51 CRCs, mean 63.1 %), L2 cluster (63 CRCs, mean 57.2 %), L3 cluster (77 CRCs, mean 50.9 %), and L4 cluster (26 CRCs, mean 40.2 %) (Fig. 2a). Notably, L3 and L4 clusters showed a significantly higher percentage of S-MSS and EO-MSS than LS-MSI and S-MSI CRC (63 and 52 % versus 35 and 21 %, respectively; *p* = 0.0002) (Table 2).

**Table 2:** Summary of the genetic and epigenetic results in the four classes of CRC

	LS-MSI	S-MSI	S-MSS	EO-MSS	*p* value
	No. of tumors/total^a^ (%)				
LINE-1 methylation					2 × 10^−5^
L1 (>60.1 %)	17/69 (25)	15/28 (54)	14/97 (14)	5/23 (22)	
L2 (54.1–60 %)	28/69 (40)	7/28 (25)	22/97 (23)	6/23 (26)	
L3 (45.8–54 %)	22/69 (32)	6/28 (21)	40/97 (41)	9/23 (39)	
L4 (<45.6 %)	2/69 (3)	0/28 (0)	21/97 (22)	3/23 (13)	
Gene methylation					10^−12^
Cluster 1	59/71 (83)	6/28 (16)	59/98 (60)	19/23 (83)	
Cluster 2	7/71 (10)	3/28 (11)	26/98 (27)	3/23 (13)	
Cluster 3	5/71 (7)	19/28 (73)	13/98 (13)	1/23 (4)	
CIN	0/9 (0)	1/6 (17)	12/16 (75)	3/6 (50)	4.9 × 10^−3^
TP53 deletion	0/9 (0)	1/6 (17)	6/16 (31)	3/6 (50)	0.10
KRAS mutation	25/58 (43)	0/26 (0)	28/94 (30)	7/23 (30)	1.2 × 10^−3^
BRAF V600E mutation	0/71 (0)	18/27 (67)	2/93 (2)	0/23 (0)	<10^−4^

*LS-MSI* lynch syndrome CRC, *S-MSI* sporadic MSI CRC, *S-MSS* sporadic MSS CRC, *EO-MSS* early onset CRC

^a^Data are not available for all cases in each subset

Univariate survival analysis on the whole series stratified by the four LINE-1 groups demonstrated that LINE-1 hypomethylation was a strong negative prognostic factor, with L4 cluster patients showing a median survival of 35 months compared to 156, 102, and more than 300 months for L3, L2, and L1 clusters, respectively (*p* = 0.0005; Fig. 2b). Survival analysis focusing specifically on each tumor class confirmed that the L4 cluster was associated with a worse prognosis when considering MSS cancers only (*p* = 0.028), while a trend toward a significant statistical value was observed when considering LS-MSI CRC only (*p* = 0.09). The small number of cases belonging to S-MSI and EO-MSS did not allow a survival analysis within these groups.

We also examined correlations of LINE-1 methylation with all the clinico-pathological features of the tumors reported in Table 1, but no significant associations were found. Interestingly, multivariable analysis of survival using the Cox proportional hazards revealed that advanced TNM stage (III and IV) and absence of MSI and LINE-1 hypomethylation (L4 cluster) were independent factors of poor prognosis (*p* = 0.0018, *p* = 0.0076 and *p* = 0.0406, respectively) (Table 3).

**Table 3:** Multivariable survival analysis

Variable	Hazard ratio	CI 95 %	*p* value
TNM stage (III–IV vs I–II)	2.39	1.37–4.13	0.0018
MSS vs MSI status	2.59	1.29–5.23	0.0076
LINE-1 hypomethylation (L4 vs L1)	2.44	1.10–5.76	0.0406

### Gene methylation profiles

Thirty-eight promoter genes were examined by Methylation-Specific Multiplex Ligation-dependent Probe Amplification (MS-MLPA) in the 220 tumor samples.

**Fig. 3:**
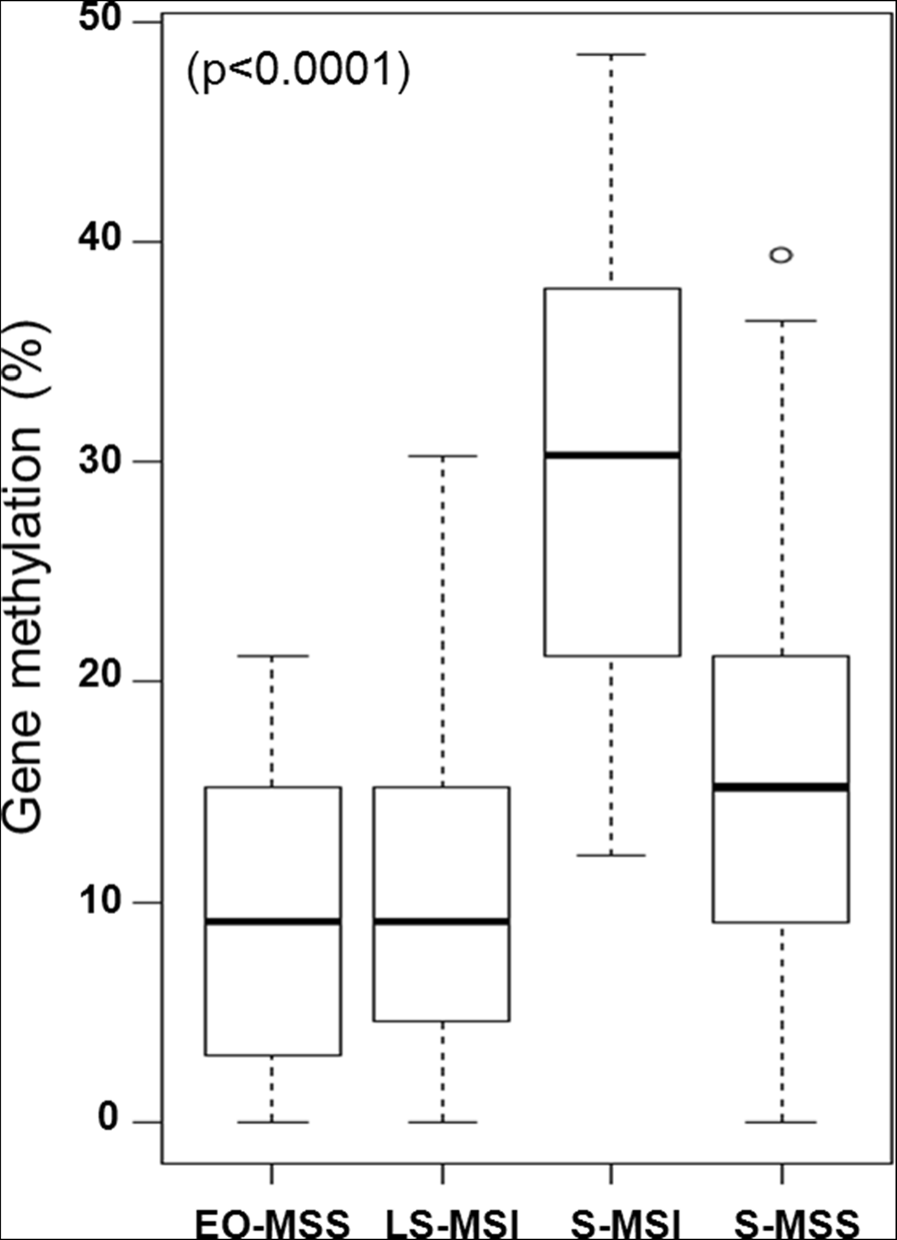
Boxplots show a significantly higher level of gene-specific methylation in S-MSI compared with those observed in EO-MSS, LS-MSI, and S-MSS

LS-MSI and EO-MSS CRCs were characterized by significantly lower levels of gene-specific methylation compared with the remaining CRCs (average methylation percentage was 8.8 % in both EO-MSS and LS-MSI versus 15 and 29 % in S-MSS and S-MSI, respectively; *p* < 0.0001). As evident from Fig. 3, gene methylation percentage was remarkably higher in S-MSI compared with the other three CRC classes (*p* < 0.0001) and it was positively correlated with MLH1 methylation (*p* < 0.0001) and with high levels of LINE-1 methylation (L1 and L2 clusters) (*p* = 0.006).

**Fig. 4:**
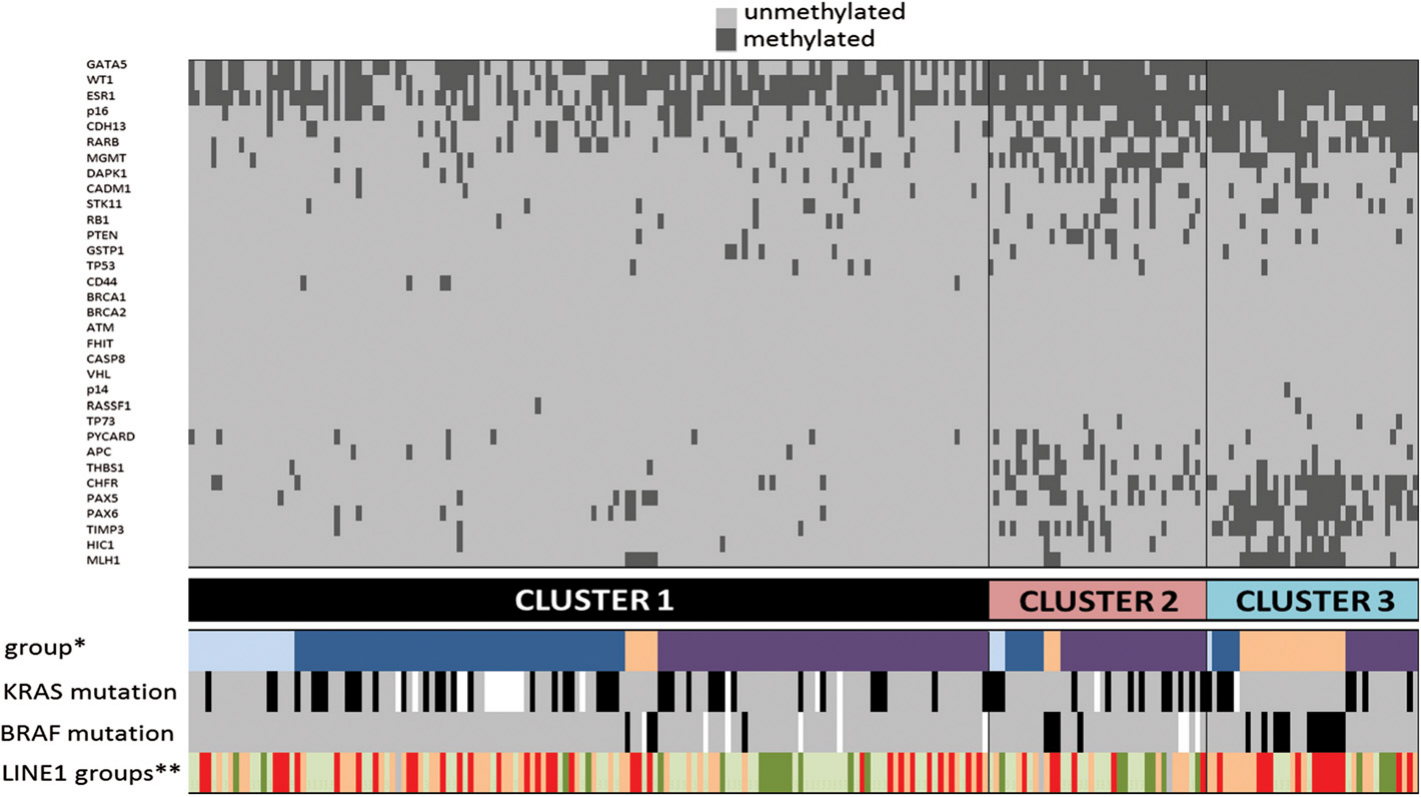
Molecular subtypes of colorectal cancer. CRC are divided into three clusters by an unsupervised hierarchical algorithm. In the bottom panel, molecular data of tumor groups (*), namely LS-MSI (*dark blue*), EO-MSS (*light blu*e), S-MSI (*orange*), and S-MSS (*purple*), are depicted. *Black* and *gray cells* indicate presence and absence of mutation, respectively; *white cells* represent data not evaluable. **: L1 (*red*), L2 (*pink*), L3 (*light green*), L4 (*green*)

Unsupervised clustering of the promoter DNA methylation profiles identified three major clusters (Fig. 4) that were characterized by distinct methylation patterns. Cluster 1 (143 patients) displayed a very homogeneous profile showing significantly lower rates of methylation compared with cluster 2 (39 patients) and with cluster 3 (38 patients) (*p* < 0.0001). In particular, these two clusters exhibited an average of 9 and 11 hypermethylated genes (24 and 29 % of gene methylation in cluster 2 and in cluster 3, respectively) and were considered as CIMP-high tumors, compared with non-CIMP cluster 1 showing an average of three hypermethylated genes (8 % of gene methylation). Cluster 1 included 83 % of EO-MSS, 83 % of LS-MSI, 60 % of S-MSS, and only 21 % of S-MSI. Cluster 1 CRCs were mainly characterized by hypermethylation restricted to only three genes that were extensively methylated in the whole series, namely GATA5, WT1, and ESR1. By contrast, cluster 2 and cluster 3 displayed extensive gene hypermethylation involving different genes. Cluster 2 was mainly composed of S-MSS (Fig. 4) and showed a higher frequency of APC methylation than cluster 3 (*p* = 0.03).

The main features of cluster 3 were the considerably higher percentage of S-MSI (68 %; *p* < 0.0001) and a specific pattern of promoter methylation. In detail, six genes including MLH1, PAX6, PAX5, RARB, CDH13, and CHFR were significantly more often methylated in cluster 3 than in cluster 2 (*p* < 0.01).

### *KRAS* and *BRAF* mutation status

*KRAS* and *BRAF* mutations were observed in 60/201 (29.8 %) and in 20/213 CRCs (9 %), respectively. As reported in Table 2, the only mutation observed in the *BRAF* gene was a V600E substitution that appeared to be closely related to S-MSI CRC (65 % of cases; *p* < 0.0001) and with a high level of gene methylation (*p* < 0.0001). In contrast, BRAF mutations were rarely observed in S-MSS (2 %) and were never observed in LS-MSI and EO-MSS CRCs.

**Fig. 5:**
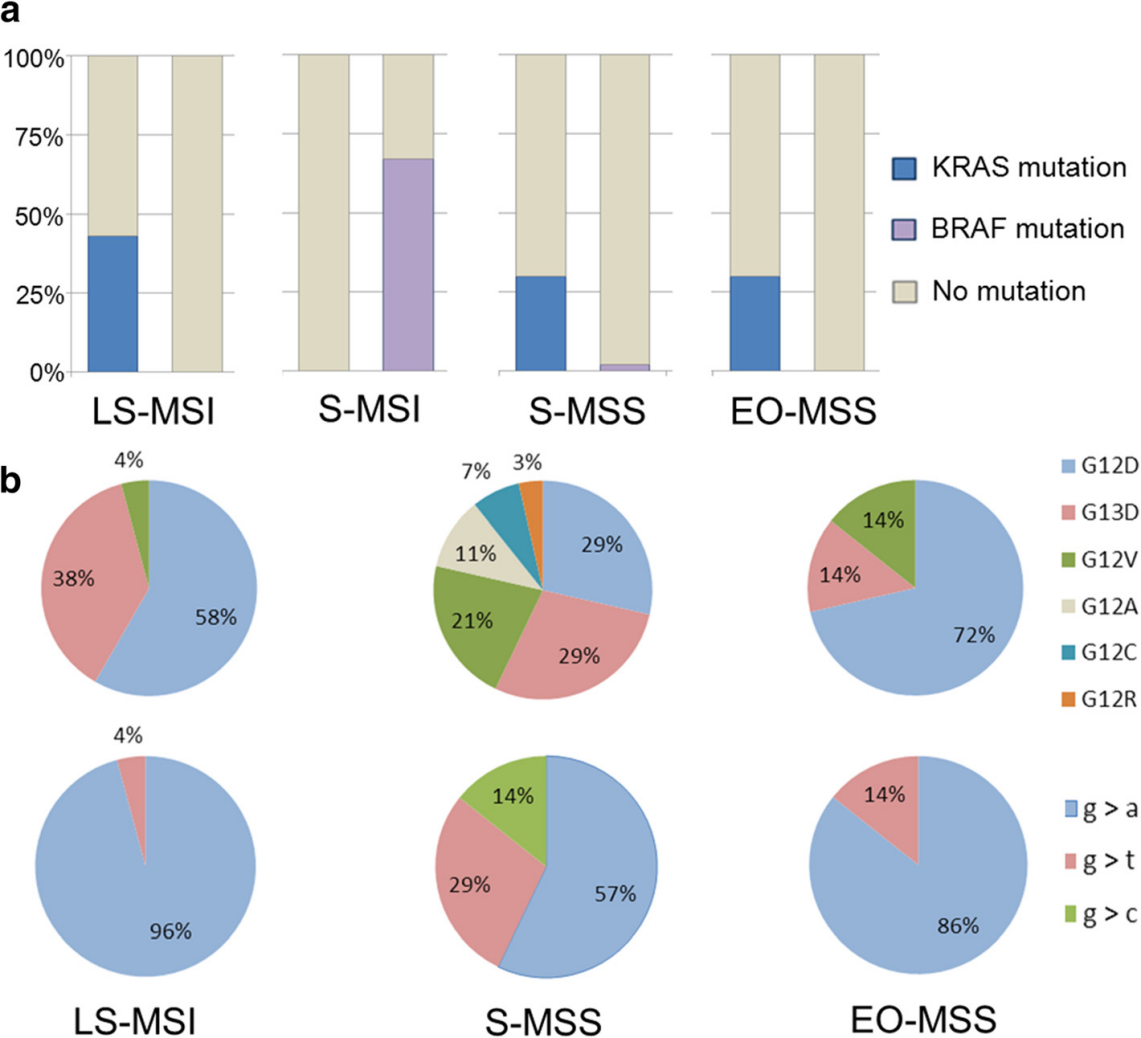
Mutation analysis in the four tumor classes. **a** Frequencies of KRAS and BRAF mutation in CRCs. **b** Distribution of the amino acidic (*upper*) and the nucleotide substitutions (*lower*) in EO-MSS, LS-MSI, and S-MSS

On the other hand, *KRAS* mutations were never detected in S-MSI, while they were identified in 43 % of LS-MSI, in 30 % of S-MSS, and in 30 % of EO-MSS CRC (*p* = 0.0012; Table 2 and Fig. 5a).

Interestingly, LS-MSI and EO-MSS CRCs showed almost exclusively G to A transition because G12D or G13D were observed in 96 and in 84 % of these tumors, respectively. *KRAS* G12V (G to T transversion) was the only additional mutation in the remaining 4 % of LS-MSI CRC and 16 % of EO-MSS CRC. Conversely, the *KRAS* mutation spectrum observed in S-MSS CRC was significantly more heterogeneous as all the substitutions G>A, G>T, and G>C were found with the following frequencies: 57, 29, and 14 %, respectively (*p* = 0.001; Fig. 5b).

### FISH results

Additional file 1: Table S1 summarizes all the fluorescent in situ hybridization (FISH) results. Gains are more frequent than losses (42 % gain and 9.9 % loss). In the majority of cases, gains of both regions on the same chromosome were detected suggesting the presence of polysomic clones in these cancers.

Monosomy, defined as the loss of both regions on the same chromosome, was observed in 10 cases. Only two regions were lost with respect to the reference probes: the TP53 locus in 10 cases (3 EO-MSS, 1 S-MSI, and 6 S-MSS; Table 2) and the 13q14 region in one case (S-MSS #59 in Additional file 1: Table S1). A CIN phenotype was observed in 16 out of 37 (43 %) CRCs. In detail, chromosomal instability was more frequently observed in S-MSS (12/16, 75 %) and in EO-MSS (3/6 cases, 50 %) (*p* = 0.10). Conversely, only one out of six (16.6 %) S-MSI and none of nine LS-MSI showed the CIN phenotype (Table 2). Interestingly, low levels of LINE-1 methylation (L3 and L4 clusters) were mainly observed in CRCs showing TP53 loss (*p* = 0.03) and a CIN phenotype (*p* = 0.08) (Additional file 1: Table S1).

## Discussion

In this research, global DNA hypomethylation as well as site-specific gene hypermethylation were examined in a multicenter series of sporadic and hereditary CRCs, using simple methods applicable to archival FFPE tissues, such as pyrosequencing and MS-MLPA. We chose to analyze a well-characterized series of inherited CRCs, including only LS patients carrying germline mutations in MMR genes. Likewise, we included in our study a selected group of patients with CRC, under the age of 40, in which the three main CRC-inherited syndromes (LS, MAP, and FAP) were excluded by genetic tests [33]. This is a crucial point since the available literature on aberrant DNA methylation in CRC is mostly focused on sporadic tumors while the role of epigenetics in hereditary and early onset CRCs is poorly known, also because specific cancer syndromes are not often accurately defined and there is no consensus about the age that defines young-onset CRC [30–32]. Actually, there is increasing attention for translation of epigenetic research into prevention and treatment of tumors [34, 35]. Aberrant methylation analyses of both sporadic and hereditary CRCs appear to be a promising strategy to better understand epigenetic mechanisms that may be generally involved in colorectal carcinogenesis regardless of the etiopathogenesis and the natural history of the tumor.

Our study revealed that the lowest levels of LINE-1 methylation (L3 and L4 in Table 2) were mainly observed in MSS tumors suggesting that this epigenetic mechanism might play a major role in CRCs without a MMR defect. Accordingly, FISH analysis demonstrated that LINE-1 hypomethylation was positively associated with *TP53* deletion (*p* = 0.03) and that a trend toward significance was observed between a CIN phenotype and the lowest levels of LINE-1 methylation (*p* = 0.08). Altogether, these findings are clearly consistent with several data sustaining a causal link between DNA hypomethylation and chromosomal instability [20–23].

As reported in Table 3, LINE-1 hypomethylation was positively associated with a poor prognosis and was an independent prognostic factor in multivariate analysis, together with TNM stage and MSI status. To the best of our knowledge, despite LINE-1 methylation having been associated with a poor clinical outcome in a long list of human tumors (see review by Baba et al., [25]), it remains controversial as to whether the LINE-1 methylation level in CRC is associated with tumor stage, and its use for prognostic purposes is still far away to being applied in clinical diagnostics. Sunami et al. [36] showed that LINE-1 demethylation is linearly correlated with TNM stage. However, in agreement with the results in our study, other recent works [27, 37] reported no relationships between the LINE-1 methylation levels and tumor stage, supporting the hypothesis that the LINE-1 hypomethylation may be initiated at an early stage of CRC remaining relatively stable throughout the long-term natural history of CRC development.

Interestingly, a subset of 24 out of 69 LS-MSI (Table 2) displayed L3 and L4 LINE-1 methylation levels and seemed to be more associated with a worse prognosis compared with the remaining LS-MSI (*p* = 0.08). Even if previous studies reported LINE-1 hypomethylation and MMR defects as two mutually exclusive markers in CRC [38], our data suggest that LINE-1 hypomethylation analysis might be useful to identify a subset of LS CRC showing a worse prognosis. In agreement with our findings, Inamura et al. [31] suggested that LINE-1 hypomethylation may be a valuable marker to find aggressive CRCs among generally indolent MSI CRCs. Our analysis extends and confirms these data in LS, suggesting a potential role of global DNA demethylation in a subset of MSI CRCs.

The role of DNA hypomethylation in EO-MSS was another point that we considered in light of recent studies reporting that a high degree of LINE-1 hypomethylation is a unique feature of young onset CRC without MSI [30]. Unlike data published by Antelo et al. [30], we did not find significantly lower levels of LINE-1 methylation in EO-MSS compared to S-MSS. In our work, EO-MSS were mainly distal (87 % of cases) and advanced CRCs (64 % of cases at stages III and IV) showing similar clinico-pathological profiles to those reported by Antelo et al. [30] and by other recent papers [39–41]. Interestingly, also the mean LINE-1 methylation levels in our series of EO-MSS were comparable to those reported by Antelo et al. [30] (54.2 ± 7.6 % versus 56.6 ± 8.6 %). By contrast, the degree of LINE-1 methylation in older onset S-MSS was very different between the two studies, being significantly lower in our study (51.7 ± 8 % versus 65.1 ± 6.3 % in the series published by Antelo et al. [30]). This observation suggests that the apparent discrepancy observed in our work is likely to be due to a different series of older onset S-MSS used in the comparative analysis and underlines the importance of the clinico-pathological and molecular features of the tumors selected for these evaluations. In conclusion, our results demonstrated comparable levels of LINE-1 methylation between EO-MSS and S-MSS, suggesting that this marker alone is not a peculiar feature of young onset CRC without MSI and it is not enough to define a distinct clinical and molecular entity of CRCs.

With regard to hypermethylation at promoters of tumor suppressor genes, our study demonstrated that S-MSI showed the highest percentages of hypermethylated genes (*p* < 0.0001), exhibiting frequent *MLH1* methylation and a specific cluster of gene methylation with respect to the remaining tumors (cluster 3 in Fig. 4). This cluster was classified as CIMP-high and showed well-known clinico-pathological and genetic features previously described for S-MSI with a CIMP phenotype [34]. Our analysis identified a second CIMP-high cluster (cluster 2 in Fig. 4) that was mainly composed of S-MSS. Cluster 2 was characterized by slightly lower levels of gene methylation compared with cluster 3 (on average 9 and 11 hypermethylated genes in cluster 2 and in cluster 3, respectively) but no other clinico-pathologic or genetic features were specifically observed in these tumors. Our analysis does not allow to conclude whether cluster 2 may be considered a subset of CRCs with intermediate levels of gene methylation (CIMP-low) as recently reported by several studies and by genome scale DNA methylation profiling [2, 13, 42]. By contrast, clustering analysis in our study has clearly distinguished a third cluster (cluster 1 in Fig. 4) that was mainly composed of young onset CRCs, including both LS-MSI and EO-MSS, that were characterized by the lowest rates of gene methylation compared with older onset CRC.

Altogether, these data are consistent with the hypotheses that aging is associated with an accumulation of aberrations in DNA hypermethylation in human tissues [43] and that S-MSI are a distinct form of epigenomic instability [15, 16] strongly associated with epigenetic silencing of *MLH1*.

An interesting result of our work is that hypermethylation of three genes, namely *ESR1*, *GATA5*, and *WT1*, was very common in all four subsets of the CRCs examined. These findings are in agreement with previous reports sustaining that the hypermethylation of these genes is a cancer-specific event in gastrointestinal carcinogenesis since the early steps of neoplastic transformation [9, 44–47]. Recently, Valo et al. [48] emphasized the early appearance of epigenetic alterations in LS associated tumorigenesis, suggesting that methylation alterations may form carcinogenic fields in histologically normal mucosa of these patients. In this context, the opportunity to investigate the environmental influences on epigenetic changes represents a new challenge to understand the role of epigenetics on CRC pathogenesis. To date, several studies reported that lifestyle, aspirin use, microbiota, and inflammation likely influence colorectal tumorigenesis via altering the local tissue microenvironment, and epigenetics plays a key role in cellular response to microenviromental change [49–51]. In summary, available literature together with our current data strongly suggest the potential utility of gene hypermethylation tests for the early detection of CRC, independently from the etiopathogenesis of the tumor.

The third issue addressed in our study was to correlate gene-specific methylation profiles with *BRAF* or *KRAS* mutations (Table 2). In agreement with previous work, we found that *BRAF* mutations were strongly associated with both *MLH1* methylations and with widespread gene hypermethylation confirming the well-established relationship of *BRAF* mutations with MSI and with a CIMP-H phenotype [14]. As expected, *BRAF* mutations were observed in 68 % of S-MSI and in only 2 % of S-MSS, whereas no *BRAF* mutations were observed in LS-MSI and in EO-MSS. This result clearly supports the use of this marker together with MLH1 methylation in order to discriminate LS-MSI from S-MSI [52] as well as confirming that the concurrence of *BRAF* mutations with a CIMP-H phenotype is a specific feature of older onset CRC [15] but was absent in EO-MSS cancers.

*KRAS* mutations were found in three classes of CRC (LS-MSI, EO-MSS, and S-MSS) and were not associated with a specific methylation profile. Interestingly, both LS-MSI and EO-MSS showed almost exclusively G:A transition, rather than G:T or G:C transversion mutations. In detail, G:A transition in cancer has been recognized as a DNA lesion caused by alkylating agents through the main mutagenic product such as *O*^6^-methylguanine (*O*^6^-meG) [53]. The high frequency of G:A transitions in LS-MSI and in EO-MSS suggests that it is a common mutagenic mechanism in these tumors, differently from S-MSS where a more heterogeneous *KRAS* mutation spectrum was observed. This finding is novel and deserves to be developed at a genome-wide level, in light of recent knowledge about specific mutation signatures in cancer and related mutagenic mechanisms [54–56].

## Conclusions

LS-MSI mainly show the absence of extensive DNA hypo- and hypermethylation, although LINE-1 hypomethylation may be observed in a subset of LS-MSI where they are associated with a worse prognosis. Genetically, they commonly display G: A transition in *KRAS* genes, an absence of a CIN phenotype and of *TP53* loss.

Among sporadic CRCs, S-MSI exhibit a specific epigenetic profile showing low rates of LINE-1 hypomethylation and widespread gene hypermethylation. These tumors are mainly characterized by *MLH1* methylation, *BRAF* V600E mutation, and absence of CIN phenotype and of *TP53* loss. By contrast, S-MSS show high frequency of LINE-1 hypomethylation and of CIN.

EO-MSS are a genetically and epigenetically heterogeneous group of CRC. Likewise LS-MSI, a subset of EO-MSS displays low rates of DNA hypo- or hypermethylation and a high frequency of G:A transition in the *KRAS* gene. On the contrary, some EO-MSS show similar features to those observed in S-MSS, such as LINE-1 hypomethylation, a CIN phenotype, and *TP53* deletion. These results indicate that a subset of EO-MSS resembles sporadic CRCs while the other subset displays some peculiar features of LS CRCs, suggesting that a genetic syndrome may not yet have been revealed in these patients.

Finally, our study confirms the potential utility of gene hypermethylation tests for the early detection of CRC and suggests that the LINE-1 methylation assay may be a useful prognostic marker in both sporadic and hereditary CRCs.

## Methods

### Clinico-pathological study

Formalin-fixed and paraffin-embedded tissue samples from 220 surgically resected hereditary and sporadic CRCs were collected from three Italian institutes, namely the Department of Pathology of the Ospedale di Circolo-University of Insubria, Varese; the Unit of Hereditary Digestive Tract Tumors, Foundation IRCCS-INT, Milan; and the Department of Diagnostic Medicine, Clinical and Public Health, University of Modena and Reggio Emilia. All CRCs were histologically reviewed at the Department of Pathology of the Ospedale di Circolo-University of Insubria, according to the World Health Organization (WHO) classification of tumors of the digestive system [57] and the TNM staging system [58]. Outcome data were collected by consulting clinical records, the Tumor Registry of the Lombardy region (Italy), and the specialized Colorectal Cancer Registry of Modena and were available for 195 patients. This study was approved by the Ethics Committee of Ospedale di Circolo di Varese (n. 0037028) and was performed according to the Helsinki Declaration.

### LINE-1 methylation study

The methylation status of LINE-1 was evaluated by bisulfite-PCR and pyrosequencing [59] in all the 220 CRCs and in twenty-five samples of histologically normal colonic mucosa. Genomic DNA was obtained from formalin-fixed and paraffin-embedded (FFPE) tissues using three representative 8-um-thick sections of each block. DNA was extracted after manual microdissection, using a QIAamp DNA FFPE tissue (Qiagen, Hilden, Germany). DNA bisulfite conversion was performed using Epitect kit (Qiagen, Hilden, Germany) according to the manufacturer’s instructions. The LINE-1 pyrosequencing assay allowed the quantification of the mean methylation percentage of four consecutive CpG sites in the LINE-1 promoter region (GenBank accession number X58075), as previously reported [60]. Fully methylated and unmethylated DNA (Millipore, Billerica MA, USA) were used as positive and negative controls in each experiment.

### Gene methylation study

Methylation analysis of a total of 38 gene promoters was performed in two replicates for each sample by MS-MLPA using the SALSA MS-MLPA ME001-tumor suppressor-1, ME002-tumor suppressor-2, and ME011-Mismatch Repair genes kit (MRC-Holland, Amsterdam, The Netherlands). MS-MLPA was performed according to the manufacturer’s instructions, and data analysis was carried out with Coffalyser software v.8 (MRC-Holland). As previously described, we fully validated the sensitivity and specificity of these MS-MLPA assays; the presence or absence of promoter methylation were scored as discrete variables using the cutoff values formerly reported [61, 62].

### *BRAF* and *KRAS* mutation analyses

Mutations in codon 600 of the *BRAF* and codon 12 and 13 of the *KRAS* gene were analyzed in duplicate by PCR-pyrosequencing using Anti EGFR MoAb response® KRAS status kit and Anti EGFR MoAb response® BRAF status kit (Diatech Pharmacogenomics, Jesi, Italy) according to the manufacturer’s instructions.

### FISH analysis

In a subset of 37 CRCs including 9 LS-MSI, 6 S-MSI, 16 S-MSS, and 6 EO-MSS, interphasic FISH was performed on sections used for conventional histologic examination (3–4 μm). The experiments were carried out as described elsewhere [63] using a panel of probes (Abbott, Chicago, USA) reported in Additional file 2: Table S2. For each FISH experiment, we used two probes mapping on to the same chromosome, one red labeled and one, the reference probe, green labeled. This FISH strategy permits the detection of loss and gain of specific regions. The cutoff values for losses and gains were defined using a panel of 10 paraffin-embedded control sections of non-neoplastic tissues and were calculated as 10 % for gain and 12 % for loss. Loss was considered when in each cell, red signals are less than green signals (reference probe); monosomy was defined when both analyzed regions on the same chromosome were loss. Gain was considered when more than two signals were observed in each cell. On the basis of these data, we defined a CIN phenotype when four or more gained regions (probes) were observed in the same colon cancer.

### Statistical analyses

Association analyses were performed using the Fisher exact test, ANOVA, and the independent sample *t* test. Supervised and unsupervised clustering analyses with k-means algorithm were used to analyze aberrant methylation data in order to distinguish different subsets of CRCs based on LINE-1 and gene methylation levels.

Patient survival was evaluated using the Kaplan-Meier method and statistically tested with the log-rank test. Patients who died within 1 month of surgery were excluded from the survival analyses. These analyses were performed with R software (https://www.r-project.org) with the mclust package [64, 65] and using GraphPad Prism V5.0 software. A *p* value ≤0.05 was considered statistically significant.

## Additional files

Additional file 1: Table S1. Interphasic FISH results of 37 colorectal cancers. (XLS 40.5 kb) Additional file 2: Table S2. Details of FISH probes used for CIN analysis. (DOCX 13.5 kb)

## Abbreviations

ANOVA: analysis of variance
CIMP-H: CpG island methylator phenotype-high
CIN: chromosomal instability
CRC: colorectal carcinoma
EO-MSS: early-onset colorectal cancer with microsatellite stability
FAP: familial adenomatous polyposis
FISH: fluorescent in situ hybridization
FFPE: formalin fixed and paraffin embedded
InSiGHT: International Society for Gastrointestinal Hereditary Tumors
LINE-1: long interspersed nucleotide element 1
LS: Lynch syndrome
LS-MSI: Lynch colorectal cancer with microsatellite instability
MMR: mismatch repair
MSI: microsatellite instability
MS-MLPA: Methylation-Specific Multiplex Ligation-dependent Probe Amplification
MSS: microsatellite stability
sCRC: sporadic colorectal carcinoma
S-MSI: sporadic colorectal cancer with microsatellite instability
S-MSS: sporadic colorectal cancer with microsatellite stability
TNM: “tumor,” “nodes,” “metastasis” (a cancer staging notation system)
WHO: World Health Organization
