# Supplementary material for: Aberrant DNA methylation profiles of inherited and sporadic colorectal cancer
Source: Clin Epigenetics. 2015 Dec 21;7:131. doi: 10.1186/s13148-015-0165-2 (PMC4687378; doi:10.1186/s13148-015-0165-2)
Supplement: Additional file 2: Table S2. — Details of FISH probes used for CIN analysis. (DOCX 13.5 kb) [file 13148_2015_165_MOESM2_ESM.docx]

| Probe | Chromosome regions |
| --- | --- |
| LSI EGR1 orange / D5S23,D5S721 green | 5p15.2 and 5q31 |
| LSI EGFR orange / CEP 7 green | 7p21 and chromosome 7 centromere |
| LSI p53 orange / CEP 17 green | TP53 and chromosome 17 centromere |
| D13S319 orange /13q34 green FISH probe kit | 13q14 and 13q34 |
| CEP 18 orange | chromosome 18 centromere |

**Table S2. Details of FISH probes used for CIN analysis.**
